# *Lactobacillus rhamnosus* GR-1 attenuates foodborne *Bacillus cereus-*induced NLRP3 inflammasome activity in bovine mammary epithelial cells by protecting intercellular tight junctions

**DOI:** 10.1186/s40104-022-00752-w

**Published:** 2022-09-09

**Authors:** Qiang Shan, Ning Liu, Xue Wang, Yaohong Zhu, Jinhua Yin, Jiufeng Wang

**Affiliations:** 1 Department of Veterinary Clinical Sciences, College of Veterinary Medicine, China Agricultural University, Beijing, 100193 China; 2 College of Animal Science and Technology, Tarim University, Alar, 843300 China

**Keywords:** *Bacillus cereus*, Intercellular tight junctions, *Lactobacillus rhamnosus* GR-1, NLRP3 inflammasome

## Abstract

**Background:**

*Bacillus cereus* is an important pathogen that causes human food poisoning, specifically diarrhea and vomiting. *B. cereus* can also induce mastitis in dairy cows and has a strong survival ability in milk, as it cannot be inactivated by high-temperature short-time pasteurization. Therefore, *B. cereus* can enter the market through pasteurized milk and other dairy products, imposing enormous hidden dangers on food safety and human health.

**Results:**

In this study, *B. cereus* 2101 (BC) was isolated from milk samples of cows with mastitis. BC grew rapidly with strong hemolysis, making it difficult to prevent mastitis and ensure food security. MAC-T cells were treated with BC and/or *Lactobacillus rhamnosus* GR-1 (LGR-1). Pretreatment with LGR-1 protected the integrity of tight junctions and the expression of zonula occludens-1 (ZO-1) and occludin destroyed by BC. Furthermore, LGR-1 pretreatment reduced the expression of NOD-like receptor family member pyrin domain-containing protein 3 (NLRP3), caspase recruitment and activation domain (ASC), Caspase-1 p20, gasdermin D (GSDMD) p30, inflammatory factors (interleukin (IL)-1β and IL-18), and cell death induced by BC. Moreover, LGR-1 pretreatment reduced NLRP3 inflammasome activity and increased expressions of ZO-1 and occludin induced by lipopolysaccharides (LPS) + ATP stimulation. MAC-T cells were transfected with NLRP3 siRNA or MCC950 and/or treated with BC and/or LGR-1. NLRP3-siRNA transfection and MCC950 attenuated BC-induced NLRP3 inflammasome activity. Expression of inflammatory cytokines and cell death suggested that the inflammatory pathway might play an important role in the induction of the NLRP3 inflammasome by BC and the protection of LGR-1.

**Conclusions:**

These results suggest that LGR-1 might be a probiotic alternative to antibiotics and could be administered to prevent mastitis in dairy cows, thus ensuring food security.

**Supplementary Information:**

The online version contains supplementary material available at 10.1186/s40104-022-00752-w.

## Introduction

Milk plays an important role in the human diet as it provides energy, proteins, and other key nutrients [1]. However, spores of *Bacillus cereus* in raw milk may not be terminated by typical pasteurization procedures using high temperature for a short time (i.e., 72 ℃ for 15 s). *B. cereus* spores can germinate and may grow to a high level in pasteurized milk and refrigerated dairy products, which induces a potential safety hazard associated with milk consumption [2]. Gao et al. collected 276 pasteurized milk samples from China and found that of these, 70 samples were contaminated with *B. cereus* [3]. A total of 26 *B. cereus* strains were isolated from 54 pasteurized whole milk samples in samples collected from supermarket chains in Wuhan, China [4]. Similarly, Chang et al. isolated 46 *B. cereus* strains from 300 pasteurized buffalo milk samples from southwestern China [5]. *B. cereus* is a Gram-positive rod-shaped bacterium, which can survive in harsh environmental conditions by producing endophytes and forming biofilms [6–8]. *B. cereus* is an important cause of food poisoning in humans and an important reason for 1.4–12% foodborne diseases worldwide [9, 10]. Food poisoning caused by *B. cereus* can manifest as emetic or diarrheal syndromes, both seriously affecting human health [10]. Numerous studies have shown that *B. cereus* may cause mastitis in dairy cows [11, 12].

*B. cereus* produces various virulence factors, including haemolysin BL (HBL), non-haemolytic enterotoxin (NHE), immune inhibitor A (InhA), cytotoxin K (Cytk), hemolysin A (HlyA), hemolysin III (HlyIII), enterotoxin FM (EntFM), and others [9, 13]. HBL and NHE can assemble on cell membranes of the host, induce pore formation, and facilitate potassium (K^+^) efflux. Subsequently, this activates the immune sensor NOD-like receptor family member pyrin domain-containing protein 3 (NLRP3) [13–15]. NLRP3 recruits apoptosis-associated speck-like proteins, which contain caspase recruitment and activation domain (ASC) and Caspase-1 and form inflammasome complexes [13, 16–18]. Activation of the NLRP3 inflammasome leads to Caspase-1 autoproteolysis, which cuts interleukin (IL)-1β, IL-18, and pro-pyroptotic factor gasdermin D (GSDMD) into their active forms [17, 19]. The active N-terminal fragment of GSDMD forms pores in the cell membrane, leading to cell death called pyroptosis [13, 20].

K^+^ efflux caused by pore-forming toxins produced by bacteria and inflammatory caspase oligomerization caused by lipopolysaccharides (LPS) produced by cell walls of Gram-negative bacteria can lead to the activation of the NLRP3 inflammasome. NLRP3 inflammasome complexes can be activated by a series of pathogen-associated molecular patterns (PAMPs) [14, 21].

As the first line of defense in mammary self-protection, bovine mammary epithelial cells are critical in the initial stage of pathogen infection [22]. Tight junctions are intercellular junctions between epithelial cells at the top region of cell–cell contacts that form an epithelial barrier and maintain epithelial polarity to protect epithelial cells [23]. The main role of this polarity is the formation of a permeability barrier, determining the selective permeability of epithelial cells [24]. The tight junction transmembrane protein occludin and the tight junction protein zonula occludens-1 (ZO-1) play an indispensable role in constituting the cytoskeleton [25]. Previous studies on probiotics showed that they could regulate the epithelial barrier function by stimulating tight junctions between epithelial cells, thereby maintaining the integrity of epithelial cells [26, 27].

*Lactobacillus rhamnosus* GR-1 (LGR-1) is the most studied female vaginal probiotic, which can inhibit the growth and prevent the adhesion of various bacterial pathogens [27]. *Lactobacilli* can protect the integrity of tight junctions by wrapping around the cell surface, thus preventing damage caused by pathogens [28]. Studies have shown that *Lactobacillus plantarum* can upregulate ZO-1 and occludin in Caco-2 cells [29, 30]. At present, most studies on LGR-1 focus on regulating the inflammatory pathway, while little attention has been paid to its protective effect on tight junctions. Studies have shown that LGR-1 can reduce *Escherichia coli*-induced Caspase-1 activation and *IL-1β* and *IL-18* production, and inhibit *E. coli*-induced pyroptosis in primary bovine mammary epithelial cells and mammary epithelial (MAC-T) cells [31, 32]. Moreover, research indicated that LGR-1 can ameliorate *E. coli*-induced damage of the cell ultrastructure, reduce the percentage of primary bovine endometrial epithelial cell apoptosis, inhibit the inflammatory response, and effectively alleviate the activation of NLRP3 inflammasome and apoptosis [33, 34]. Further studies have shown that LGR-1 inhibits biofilm formation on *Candida albicans* strains on abiotic surfaces and *Salmonella enterica serovar Typhimurium* [35, 36]. The purpose of this experiment was to explore LGR-1 attenuation of *B. cereus-*induced intercellular tight junction damage and NLRP3 inflammasome activity in bovine MAC-T cells.

## Materials and methods

### MAC-T culture

MAC-T cells were resuscitated and passaged three times before the experiment. The cells were then incubated in DMEM/Ham’s F-12 medium (GE Healthcare Life Sciences HyClone Laboratories, Utah, USA) supplemented with 10% fetal bovine serum (Thermo Fisher Scientific, Rockford, Waltham, MA, USA), and 100 U/mL of penicillin and streptomycin (Invitrogen, Carlsbad, CA, USA) at 37 ℃ in an atmosphere of 5% CO_2_.

### Isolation and culture of *B. cereus*

*B. cereus* 2101 (BC) was isolated from milk samples of dairy cows with mastitis from Youran dairy Co., Ltd. (Daqing, China). Subsequently, the strain was sent to Beijing Tianyi Huiyuan Biotechnology Co., Ltd. (Beijing, China) for bacterial 16S rRNA sequencing. The sequence was subjected to Nucleotide BLAST alignment on NCBI [37] to obtain strain information. BC was grown in Luria–Bertani (LB) broth (AOBOX, Beijing, China) overnight under aerobic conditions at 37 ℃ and shaking at 200 r/min. This procedure was used to stimulate MAC-T for 3 h with a multiplicity of infection (MOI) of 5.

### *Lactobacillus rhamnosus* GR-1 culture

ATCC 55826 LGR-1 was purchased from the American Center for the Preservation of Typical Cultures (Manassas, VA, USA) and grown at 37 ℃ for 24 h in de Man, Rogosa, and Sharpe (MRS) broth (AOBOX, Beijing, China) under microaerophilic conditions. After overnight incubation, LGR-1 was diluted into 1:100 with MRS broth and subcultured for about 12 h until the mid-logarithmic phase (i.e., optical density at 600 nm (OD_600_) = 0.5). This was used to pretreat MAC-T cells for 3 h with a MOI of 100.

### Biosecurity statement

Clinical isolates of *B. cereus* were treated strictly in accordance with the Regulations on biological safety of Pathogen Microbiology laboratory by the State Council of the People’s Republic of China (000014349/2004–00195). All necessary safety operations were implemented to avoid pathogen transmission and infection.

### Lactate dehydrogenase assay

To examine cell death under different treatments, lactate dehydrogenase (LDH) levels were measured using the LDH Cytotoxicity Assay Kit (Beyotime Biotechnology, Shanghai, China) according to the manufacturer’s instructions. The cell culture plate was centrifuged at 400 × *g* for 5 min, and 120 μL supernatant of each well was added to the corresponding wells of a new 96-well plate. Then, 60 μL LDH detection solution was added to each well. Supernatant and LDH detection solution were mixed and incubated in the dark at room temperature for 30 min. The absorbance was measured at 490 nm. The LDH level released by uninfected cells was used as control, and LDH release reagent was added to cells to obtain the maximum enzyme activity (max). The percentage of LDH released was calculated using the following equation: [(LDH_infected_−LDH_uninfected_)/(LDH_max_−LDH_uninfected_)] × 100.

### Determination of growth curves

Both 1 mL LB broth and BC solution were added to sterilized turbidity tubes, and turbidity was adjusted to 0.2 McFarland Standard (MCF) by turbidity meter (BioMérieux, Durham, NC, USA). A total of 198 μL of LB broth was added to each well of a 96-well plate and then, 2 μL of BC solution with a turbidity of 0.2 was also added to each well. The experiment was repeated three times. The plate was placed in a microplate reader (Tecan, Switzerland) and OD_600_ was measured every hour continuously for a 31-h measurement.

### Strain characteristics

BC was cultured on LB nutrient agar (AOBOX, Beijing, China) supplemented with 5% sheep blood (Solarbio, Beijing, China), and incubated upside down overnight at 37 ℃. BC was differentially stained according to the instructions provided with the Gram Staining Kit (Solarbio, Beijing, China).

### Virulence factors of *B. cereus* 2101

The DNA of BC was extracted with TIANamp Bacterial DNA Kit (TIANGEN, Beijing, China). Briefly, 5 mL bacterial solution was centrifuged at 11,500 × *g* for 1 min after adding lysozyme and RNase A at 37 °C for 30 min. The treated liquid was added to the DNA adsorption column for recovery, and the recovered DNA was assessed for concentration and purity by NanoDrop® ND-2000C (Thermo Fisher Scientific, USA). Virulence gene primers (Table 1) were used for PCR virulence gene detection [38, 39]. PCR products were analyzed by 1% agarose gel electrophoresis for 30 min at 125 V in 1 × Tris–acetate-EDTA buffer, stained with ethidium bromide and photographed under UV transillumination.

**Table 1:** Primer sequences of virulence genes

Gene name	Direction	Primer sequences (5’ to 3’)	Length, bp
*HBLA*	F	CGGGATCCGCAGTCATACCAATAGAAACTTTTGC	1351
	R	CCCTCGAGTCAGTTCATTATATTTTGTACTTTGTCTTTATACAC	
*HBLB*	F	CGGAATTCTCACCAGTAACAACTTTTGCAAGTGAA	1072
	R	CCCTCGAGCTATTTTTGTGGAGTAACAGTTTCCACTTTT	
*HBLD*	F	CGGGATCCGCATTTGCACAAGAAACGACCG	1156
	R	CCCTCGAGCTACTCCTGTTTAAAAGCAATATCTTTTGAAATGAA	
*NHEA*	F	CGGGATCCACGAGTTGCTTCATTCCTGTAAGC	1132
	R	CCCTCGAGTTAATGTACTTCAACGTTTGTAACGTAATCTTCAAAT	
*NHEB*	F	CGGAATTCAATATTATGCCGGCTCATACGTATGCA	1162
	R	CCCTCGAGTTATGCTTTTTTCGTATCTACTACTTTAATATCTTC	
*NHEC*	F	CGGAATTCATGCCGGCTCATACGTAT	1156
	R	CCCTCGAGTTATGCTTTTTTCGTATCTACTACTTTAATATCTTCA	
*InhA*	F	CGGAATTCATGAGTGCTCCGTTAGCATATGCA	2344
	R	CCCTCGAGTTAACGTTTAATCCAAACAGCGCCTGC	
*Cytk*	F	CGGAATTCCCTGCTACTTACGCTCAAAC	949
	R	CCCTCGAGTTATTTTTTCTCTACTAATTTCTTATTCTTCCAATCTAG	
*HlyA*	F	CGGAATTCGCCATTATGGCCGGACT	778
	R	CCCTCGAGTTATTCCCCTTTCCCTTTTTGTTTTAG	
*HlyIII*	F	CGGAATTCGCAATTACACATGGTATCGGTG	619
	R	CCCTCGAGTTATGCTGTAGGTAAGACATAAAAGAGTACA	
*EntFM*	F	ATGAAAAAAGTAATTTGCAGG	1269
	R	TTAGTATGCTTTTGTGTAACC	

*HBLA*, *HBLB*, *HBLD*, haemolysin BL A, B, D; *NHEA*, *NHEB*, *NHEC*, non-haemolytic enterotoxin A, B, C; *InhA*, immune inhibitor A; *Cytk*, cytotoxin K; *HlyA*, hemolysin A; *HlyIII*, hemolysin III; *EntFM*, enterotoxin FM

### Determination of minimum inhibitory concentration

The minimum inhibitory concentration (MIC) of BC was determined by the broth microdilution method using *E. coli* ATCC 25922 as quality control reference. The experiment was operated strictly according to performance standards for antimicrobial susceptibility testing M100 of the clinical and laboratory standards institute [40]. The results of MIC for ampicillin (AMP), amoxicillin (AMC), azithromycin (AZI), cefazolin (CZ), ciprofloxacin (CIP), gentamicin (GM), meropenem (MEM), kanamycin (KAN), streptomycin (STR), and tetracycline (TE) were examined. Three independent repetitions were performed per trial. Based on the MIC for each antimicrobial agent, the isolate was defined as “susceptible (S)”, “intermediate (I)”, or “resistant (R)”.

### Drug treatments

MAC-T cells were treated under different conditions as follows: (i) control group without treatment (CONTROL); (ii) exposure to BC for 3 h (BC); (iii) pretreatment with LGR-1 for 3 h and exposure to BC for 3 h (LGR-1 + BC); (iv) treatment with LGR-1 for 3 h (LGR-1); (v) pretreatment with MCC950 (100 nmol/L, HY-12815, MedChemExpress, USA) for 30 min and exposure to BC for 3 h (MCC950 + BC); (vi) treatment with MCC950 for 30 min (MCC950); (vii) pretreatment with LGR-1 for 3 h, then priming using LPS from *E. coli* 0111: B4 (500 ng/mL, L4391, Sigma-Aldrich, USA) for 4 h and stimulation with ATP (5 mmol/L, A6419, Sigma-Aldrich, USA) for 45 min (LGR-1 + LA); (viii) priming using LPS for 4 h and stimulated with ATP for 45 min (LA); (ix) pretreatment with increasing KCl concentrations of 5 mmol/L, 25 mmol/L, 50 mmol/L, and 75 mmol/L (P9541, Sigma-Aldrich, USA) for 30 min and exposure to BC for 3 h; (x) pretreatment with 50 mmol/L of KCl and priming using LPS for 4 h and stimulation with ATP for 45 min; (xi) exposure to BC (MOI = 0.5, 5, or 50) for 3 h; (xii) pretreatment with LGR-1 (MOI = 1, 10, 100, or 1000) for 3 h, and then exposure to BC (MOI = 5).

### Transmission electron microscopy

At 3 h after BC challenge, MAC-T cells were harvested and fixed in 3% glutaraldehyde (pH = 7.4). Samples were treated following standard transmission electron microscopy (TEM) procedures [31].

### Western blotting

MAC-T cells were lysed in lysis buffer, composed of 1 mL of RIPA buffer and 10 μL of phenylmethanesulfonyl fluoride (Solarbio, Beijing, China). The resulting lysates were centrifuged at 13,000 × *g* for 10 min at 4 ℃ to pellet insoluble material, and supernatants were used for Western blotting analysis. Protein concentrations were determined using a BCA Protein Assay kit (Thermo Fisher Scientific, Waltham, MA, USA). The following primary antibodies were used: ZO-1 (21773–1-AP, 1:1000 dilution, Proteintech Group), occludin (27260–1-AP, 1:1000 dilution, Proteintech Group), NLRP3 (19771–1-AP, 1:500 dilution, Proteintech Group), ASC (10500–1-AP, 1:1000 dilution, Proteintech Group), Caspase-1 (sc-56036, 1:200 dilution, Santa Cruz Biotechnology), GSDMD (20770–1-AP, 1:2000 dilution, Proteintech Group), and mouse anti-glyceraldehyde-3-phosphate dehydrogenase (GAPDH) mAb (60004–1-Ig, 1:5000 dilution, Proteintech Group). Horseradish peroxidase-conjugated affinipure goat anti-mouse IgG (H + L) (SA00001-1, 1:8000 dilution, Proteintech Group) or goat anti-rabbit IgG (H + L) (SA00001-2; 1:8000 dilution, Proteintech Group) were used as secondary antibodies. The Tanon 6200 chemiluminescence imaging workstation (Tanon, Shanghai, China) was used to visualize immunoreaction bands. Densitometric values of Western blotting images were obtained from three independent experiments using Image J software (National Institutes of Health, Bethesda, MD, USA). Results are presented as the ratio of ZO-1, occludin, NLRP3, ASC, GSDMD, or Caspase-1 band intensity to that of GAPDH.

### Immunofluorescence

Cells were fixed with 4% paraformaldehyde, treated with 1% (v/v) Triton X-100, and then blocked with 2% BSA. MAC-T cells were incubated with NLRP3 (1:100 dilution), ASC (1:100 dilution), GSDMD (1:200 dilution), Caspase1 (1:50 dilution), and ZO-1 (1:750 dilution) at 4 ℃ overnight. Then, samples were incubated with secondary antibody at room temperature for 1 h. DAPI (C0065, Solarbio, Beijing, China) was used for nuclear staining. MAC-T cells were observed and photographed using a Nikon A1 confocal laser scanning microscope.

### Quantitative real-time PCR

RNAiso Plus (9108, TaKaRa, Japan) was used to extract total RNA from MAC-T cells and the PrimeScriptTM RT Reagent Kit (RR047A, TaKaRa, Japan) was used for RNA transcription. SYBR Green PCR Master Mix (LS2062, Promega, USA) was used for real-time quantitative PCR (qPCR). Target gene primers (Table 2) were designed by Primer BLAST [41] according to the relevant literature or the CDS region sequence of the target gene in GenBank, and synthesized by Sangon Biotech Co., Ltd. (Shanghai, China). The mRNA expressions of *IL-1β*, *IL-18*, *NLRP3*, *ZO-1*, and occludin were normalized to the mRNA expression of *GAPDH*. All primer sequences are shown in Table 2 and the gene expression levels were analyzed with the 2^−ΔΔCT^ method.

**Table 2:** Primers sequences of qPCR

Gene name	Direction	Primer sequences (5’ to 3’)	Concentration, μmol/L	Reaction efficiency, %
*IL-1β*	F	CCTCGGTTCCATGGGAGATG	10	93.6
	R	AGGCACTGTTCCTCAGCTTC	10	
*IL-18*	F	TCAGATAATGCACCCCAGACC	10	91.3
	R	GATGGTTACGGCCAGACCTC	10	
*NLRP3*	F	ATGGTAAGTGTCCGCTGCAA	10	95.5
	R	ATGCCTTCTCTTCCCCGTTG	10	
*ZO-1*	F	CACTATGACCCCGAGGAGGA	10	98.1
	R	AACTGTGGCTGAGATTGGGG	10	
Occludin	F	CCATCGGAGTTTCAGGTGAATG	10	91.2
	R	CTCCGCCTGAAGAAGCAGAAA	10	
*GAPDH*	F	GTCTTCACTACCATGGAGAAGG	10	94.4
	R	TCATGGATGACCTTGGCCAG	10	

*IL-1β*, *IL-18,* Interleukin 1β, 18, *NLRP3,* NOD-like receptor family member pyrin domain-containing protein 3, *ZO-1,* zonula occludens-1

### RNA interference

To verify the importance of the NLRP3 inflammatory pathway in *B. cereus*-infected MAC-T cells, NLRP3 was knocked out by gene silencing. MAC-T cells were seeded in 6-well plates. When MAC-T cells had grown to 60–80% confluence, NLRP3-siRNA (si-NLRP3, 50 pmol/mL, Sense 5’-GGAGAGACCUUUAUGAGAATT-3’, Antisense 5’-UUCUCAUAAAGGUCUCUCCTT-3’, GenePharma, Suzhou, China) diluted with Lipo8000™ Transfection Reagent (Beyotime Biotechnology, Shanghai, China) was transfected into MAC-T cells. After transfection for 5 h, the medium was replaced with fresh medium and incubated at 37 °C for 48 h for subsequent treatment and analysis.

### Statistical analysis

All statistical analyses were performed using GraphPad Prism 9.2.0. The *t*-test was used for comparisons between two groups, and one-way ANOVA was used for comparisons between three or more groups. Values are expressed as means ± SEM. *P < 0.05, **P < 0.01, and ***P < 0.001 were considered indicative of statistical significance. All experiments were performed in triplicate.

## Results

### Screening and characteristics of BC

**Fig. 1:**
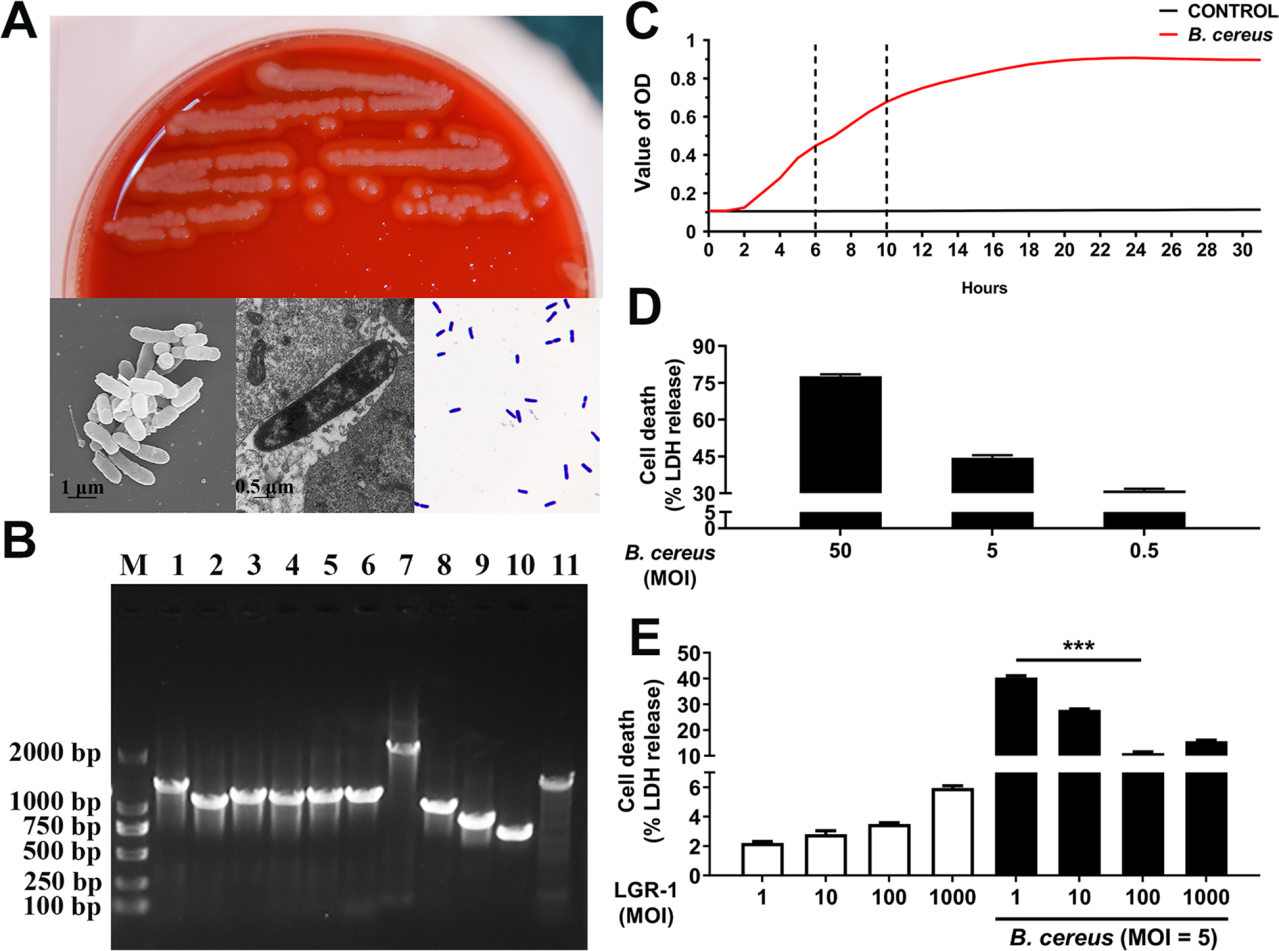
*Lactobacillus rhamnosus* GR-1 (LGR-1) alleviated *Bacillus cereus* 2101 (BC)*-*induced cell death. MAC-T cells were treated with BC (MOI = 0.5, 5 or 50) and/or LGR-1 (MOI = 1, 10, 100, or 1000) for 3 h. (**A**) The blood plate shows that the strain was hemolytic. Transmission electron microscopy (TEM) showed that the strain was rod-shaped. The scale bar is shown in the lower left corner. The color was purple after staining with the Gram Staining Kit. (**B**) Virulence factors of BC. M. Marker; 1. *HBLA*; 2. *HBLB*; 3. *HBLD*; 4. *NHEA*; 5. *NHEB*; 6. *NHEC*; 7. *InhA*; 8. *CytK*; 9. *HlyA*; 10. *HlyIII*; 11. *EntFM*. (**C**) Growth curves of BC. (**D**) Cell death induced by BC. (**E**) Cell death induced by LGR-1 and/or BC. One-way ANOVA was used for comparison between groups. The data are mean ± SEM of three independent experiments. *** *P* < 0.001

BC was isolated and identified from milk samples of dairy cows with mastitis. BC was identified as a bar-like Gram-positive bacterium with hemolytic activity (Fig. 1A). *HBLA*, *HBLB*, *HBLD*, *NHEA*, *NHEB*, *NHEC*, *InhA*, *HlyA*, *HlyIII*, *CytK*, and *EntFM* were detected (Fig. 1B). The growth curve of BC showed that the logarithmic phase lasted between 6 and 12 h (Fig. 1C). BC with MOI = 50 and MOI = 5 showed strong cytotoxicity for MAC-T cells (Fig. 1D). MIC indicated that BC was resistant to AMP, AMC, AZI, CZ, CIP, STR, and TE (Table 3), and had multi-antibiotic resistance. Pretreatment with LGR-1 (MOI = 100) for 3 h could effectively alleviate cell death after exposure to BC (MOI = 5) for 3 h (Fig. 1E).

**Table 3:** Antimicrobial susceptibility profiles of *B. cereus* clinical isolates^a^

Strains	**Antibiotics**^b^									
	AMP	AMC	AZI	CZ	CIP	GM	MEM	KAN	STR	TE
*B. cereus* 2101	R	R	R	R	R	S	S	S	R	R

^a^Isolates were defined as “susceptible (S)”, “intermediate (I)”, or “resistant (R)” based on MIC for each antimicrobial agent

^b^Ampicillin (AMP), amoxicillin (AMC), azithromycin (AZI), cefazolin (CZ), ciprofloxacin (CIP), gentamicin (GM), meropenem (MEM), kanamycin (KAN), streptomycin (STR), and tetracycline (TE)

### LGR-1 protects BC-damaged intercellular tight junctions

**Fig. 2:**
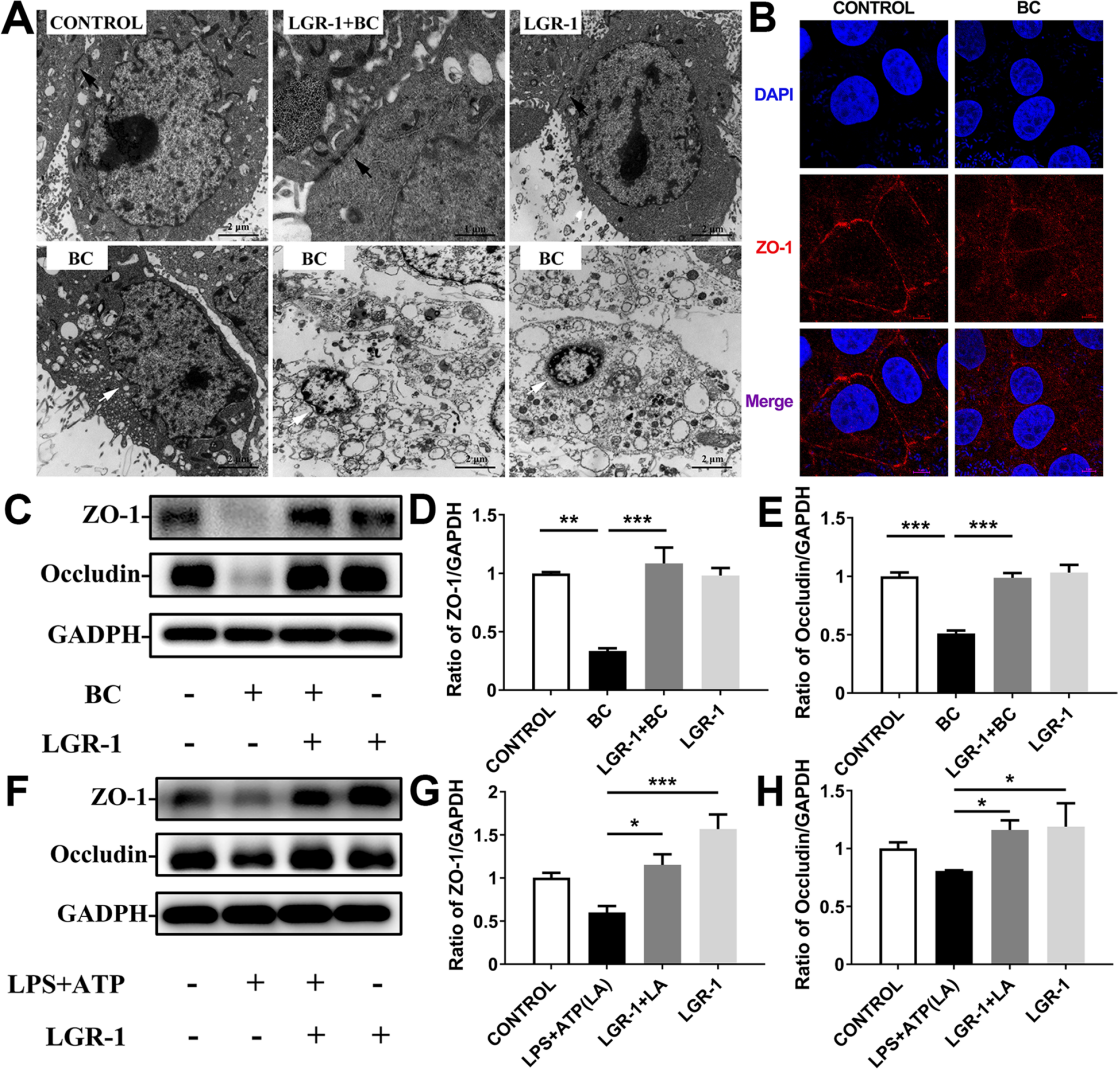
LGR-1 protects BC damaged intercellular tight junctions. MAC-T cells were treated with BC (MOI = 5) or LPS (500 ng/mL) + ATP (5 mmol/L) and/or LGR-1 (MOI = 100). (**A**) The ultrastructure of LGR-1 and/or BC treated cells was visualized by TEM. The scale bar is shown in the lower right corner. (**B**) Expression of ZO-1 in cells measured by immunofluorescence analysis; the scale bar is shown in the lower right corner. (**C**) and (**F**) Protein levels of ZO-1 and occludin in MAC-T. (**D**) and (**G**) Relative protein level of ZO-1. (**E**) and (**H**) Relative protein level of occludin. The data for the CONTROL group were used to normalize the data of each treatment group. White arrows indicate the nuclear membrane and black arrowheads indicate junctional complexes. Comparisons among groups were calculated using one-way ANOVA. Data are means ± SEM of three independent experiments. *P < 0.05, **P < 0.01, and ***P < 0.001

Tight junctions are a key structure of the epithelial barrier. They are composed of tight junction proteins, which can protect epithelial cell integrity. TEM showed that the tight junction structures of the CONTROL group, LGR + BC group, and LGR-1 group were complete, while the tight junction structure of BC group was destroyed (Fig. 2A). Immunofluorescence showed that the ZO-1 protein in the CONTROL group, LGR + BC group, and LGR-1 group was distributed between cells, and the structure was complete, while the integrity of the ZO-1 protein in the BC group was destroyed (Fig. 2B, Additional file 1: Fig. S1). Western blotting and qPCR results further support this phenomenon that compared with the CONTROL group, the BC group had significantly decreased (*P* < 0.01) protein and mRNA expression of *ZO-1* and occludin, while the LGR-1 + BC group had significantly increased (*P* < 0.001) protein and mRNA expression compared with BC group. This indicates that pre-treatment with LGR-1 can prevent BC to destroy tight junction proteins (Fig. 2C–E, Fig. S1).

### LGR-1 attenuated BC-induced inflammatory responses

**Fig. 3:**
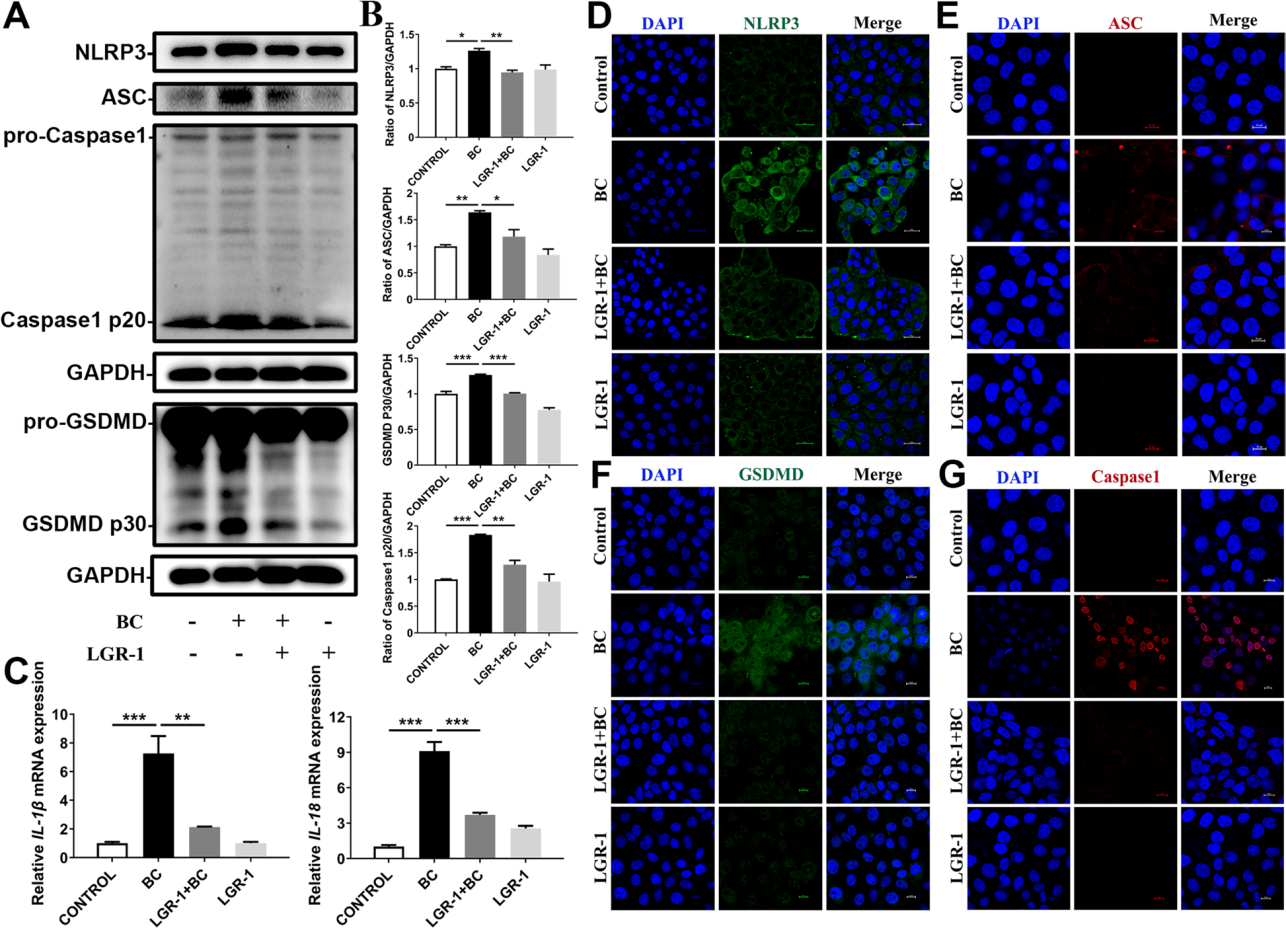
LGR-1 attenuated BC-induced NLRP3 inflammasome activity. MAC-T cells were treated with BC (MOI = 5) and/or LGR-1 (MOI = 100) for 3 h. (**A**) and (**B**) Protein levels of NLRP3, ASC, Caspase-1 p20, GSDMD p30. (**C**) *IL-1β* and *IL-18* mRNA level. Expression of NLRP3, ASC, GSDMD, and Caspase-1 in cells measured by immunofluorescence analysis; scale bar shown in the lower right corner. (**D**) NLRP3. (**E**) ASC. (**F**) GSDMD. (**G**) Caspase-1. The data for the CONTROL group were used to normalize the data of each treatment group. Comparisons among groups were calculated using one-way ANOVA. Data are means ± SEM of three independent experiments. *P < 0.05, **P < 0.01, and ***P < 0.001

TEM further showed membranolysis, loss of cytoplasmic content, chromosome condensation, and chromatin margination in BC-stimulated MAC-T cells (Fig. 2A). Western blotting analysis showed that the protein expressions of NLRP3, ASC, Caspase-1 p20, and GSDMD p30 increased in the BC group compared with the CONTROL group, indicating that NLRP3 inflammasome activity was induced by BC stimulation (Fig. 3A and B, *P* < 0.05). The expressions of these proteins in the LGR-1 + BC group were significantly lower compared with the BC group (Fig. 3A and B). Further, immunofluorescence was used to explore the effect of LGR-1 on the BC-induced NLRP3 inflammasome activity. BC formed the ASC speck, which is a hallmark of NLRP3 inflammasome activity (Fig. 3E) [42]. The expression levels of NLRP3, GSDMD, and Caspase-1 were increased in the BC group, and were decreased in the LGR-1 + BC group (Fig. 3D, F, and G ). Similarly, the expressions of the NLRP3 pathway target genes *IL-1β* and *IL-18* were also increased in the BC group, while pre-treatment with LGR-1 attenuated the BC-induced expression of *IL-1β* and *IL-18* (Fig. 3C, *P*< 0.01).

### BC-induced NLRP3 inflammasome activity requires K^+^ efflux

**Fig. 4:**
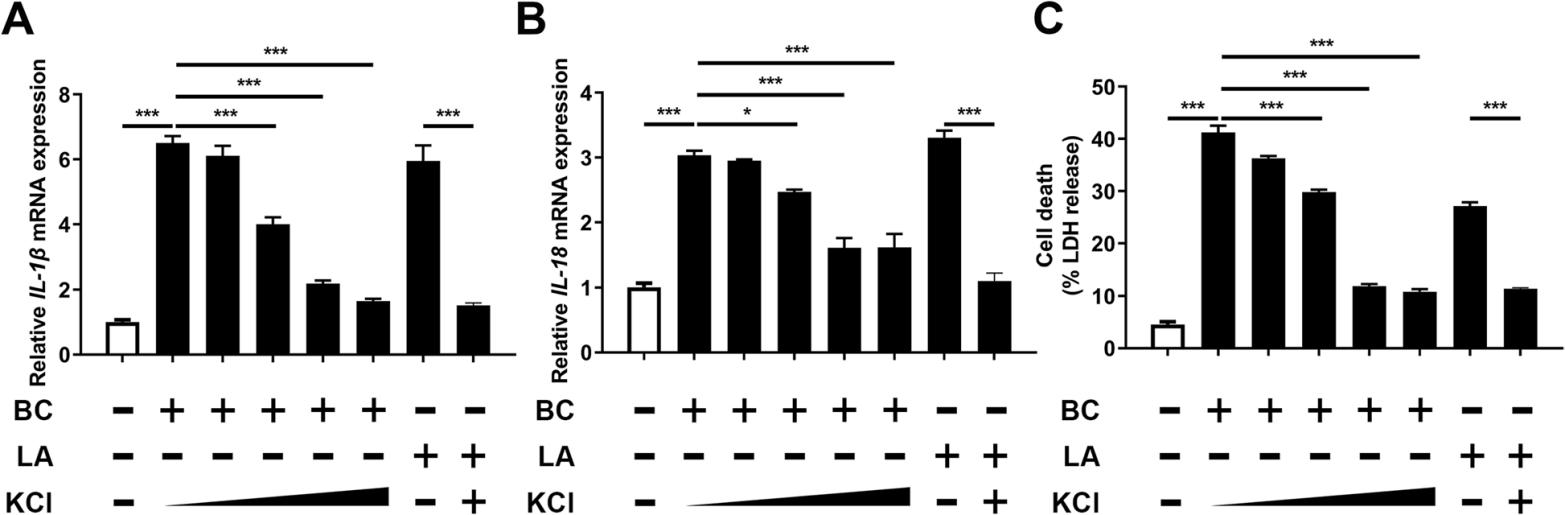
BC-induced NLRP3 inflammasome activity requires K^+^ efflux. MAC-T cells were treated with BC (MOI = 5) or LPS (500 ng/mL) + ATP (5 mmol/L) in the absence (-) or presence ( +) of 50 mmol/L KCl ( +) or at increasing concentrations of KCl (5, 25, 50 and 75 mmol/L). (**A**) *IL-1β* mRNA level. (**B**) *IL-18* mRNA level. (**C**) Cell death in MAC-T. The data for the CONTROL group were used to normalize the data of each treatment group. Comparisons among groups were calculated using one-way ANOVA. Data are means ± SEM of three independent experiments. *P < 0.05 and ***P < 0.001

The efflux of K^+^ efflux has been identified as the central mechanism for the activation of NLRP3 inflammasome [21, 43]. In this study, extracellular K^+^ efflux inhibited the NLRP3 inflammasome activity induced by BC in a dose-dependent manner, and effectively inhibited the expressions of *IL-1β* and *IL-18* and cell death at 50 mmol/L K^+^ efflux (Fig. 4A–C, *P* < 0.05). The appropriate addition of extracellular K^+^ efflux also inhibited the NLRP3 inflammasome activity by LPS + ATP, which was consistent with the above results.

### LGR-1 inhibits inflammatory responses by protecting tight junctions

**Fig. 5:**
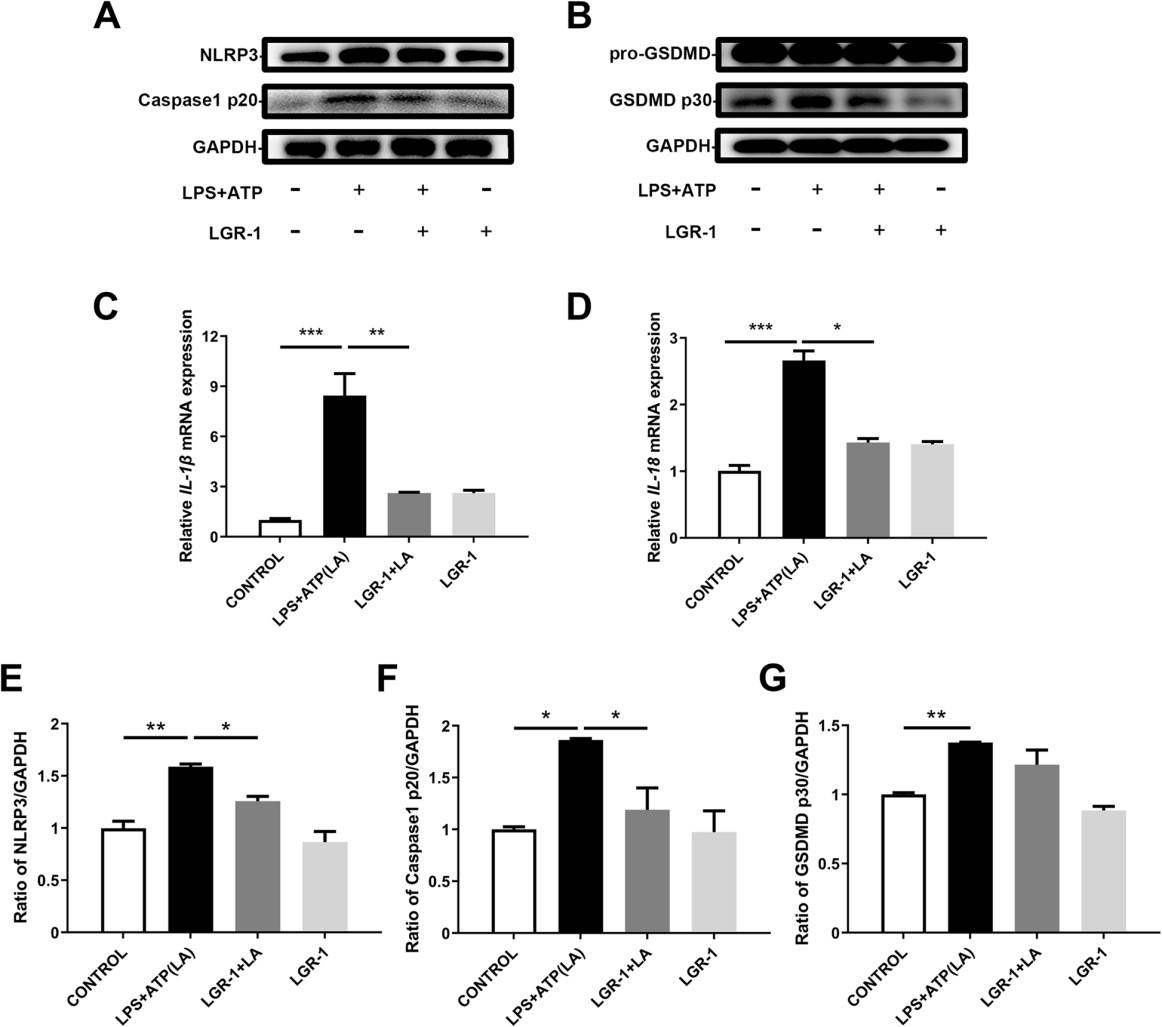
LGR-1 attenuated LPS + ATP-induced NLRP3 inflammasome activity. MAC-T cells were treated with LPS (500 ng/mL) + ATP (5 mmol/L) and/or LGR-1 (MOI = 100). (**A**) Protein levels of NLRP3 and Caspase-1 p20. (**B**) Protein levels of GSDMD p30. (**C**) *IL-1β* mRNA level. (**D**) *IL-18* mRNA level. (**E**) Relative protein level of NLRP3. (**F**) Relative protein level of Caspase-1 p20. (**G**) Relative protein level of GSDMD p30. The data for the CONTROL group were used to normalize the data of each treatment group. Comparisons among groups were calculated using one-way ANOVA. Data are means ± SEM of three independent experiments. *P < 0.05, **P < 0.01, and ***P < 0.001

LGR-1 attenuated BC-induced NLRP3 inflammasome activity and significantly reduced NLRP3 inflammasome activity and expressions of *IL-1β* and *IL-18* caused by LPS + ATP (Fig. 5A–F, *P* < 0.05). Treatment with LGR-1 alone did not affect the NLRP3 inflammatory pathway. Thus, tight junction proteins were explored, showing that when the NLRP3 pathway was stimulated by LPS + ATP, pre-treatment with LGR-1 significantly increased the protein contents of ZO-1 and occludin (Fig. 2F–H, *P *< 0.05).

### BC-induced inflammatory responses by NLRP3

**Fig. 6:**
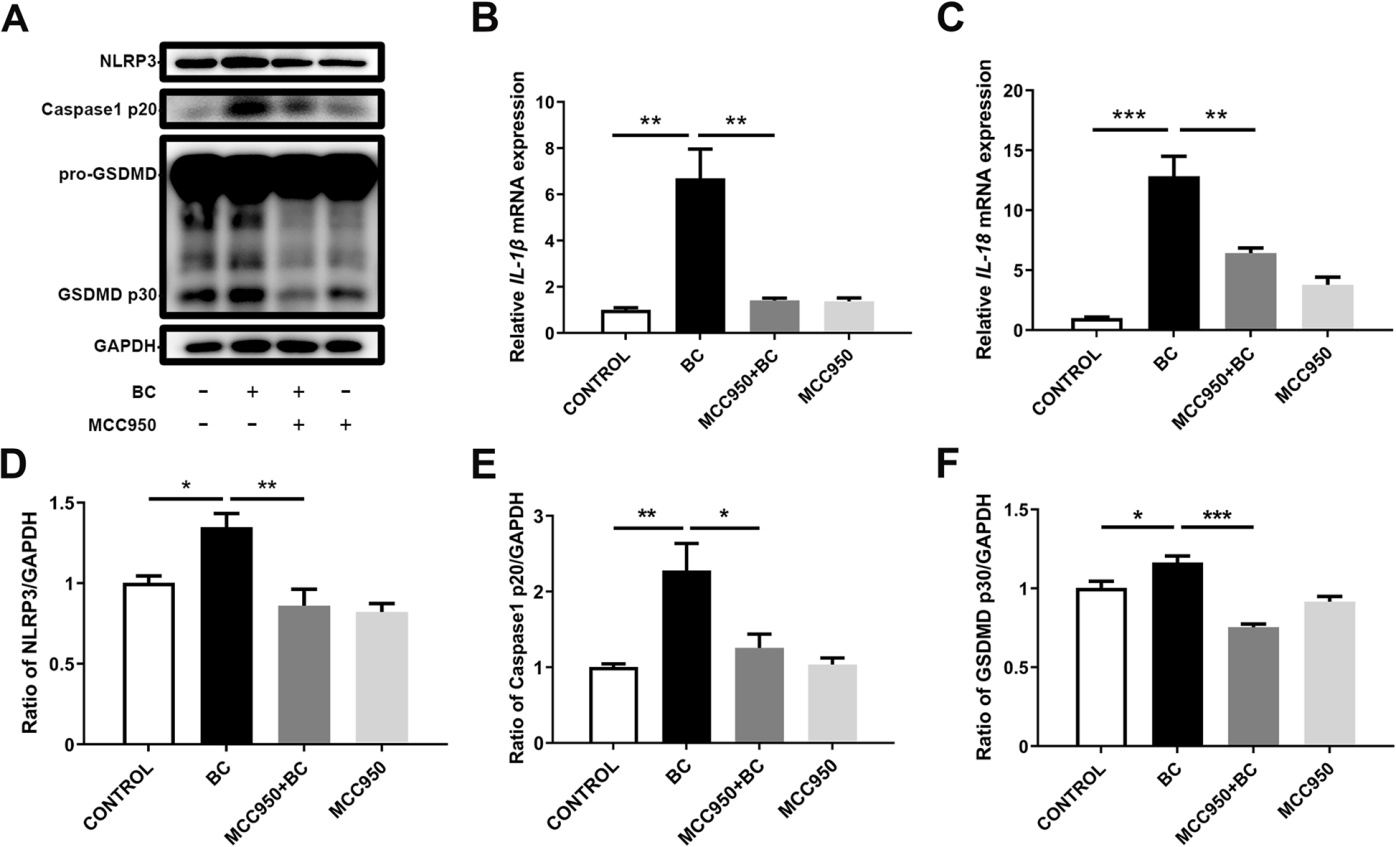
BC induced NLRP3 inflammasome activity. MAC-T cells were treated with BC (MOI = 5) and/or MCC950 (100 nmol/L). (**A**) Protein levels of NLRP3, Caspase-1 p20, GSDMD p30. (**B**) *IL-1β* mRNA level. (**C**) *IL-18* mRNA level. (**D**) Relative protein level of NLRP3. (**E**) Relative protein level of Caspase-1 p20. (**F**) Relative protein level of GSDMD p30. The data for the CONTROL group were used to normalize the data of each treatment group. Comparisons among groups were calculated using one-way ANOVA. Data are means ± SEM of three independent experiments. *P < 0.05, **P < 0.01, and ***P < 0.001

**Fig. 7:**
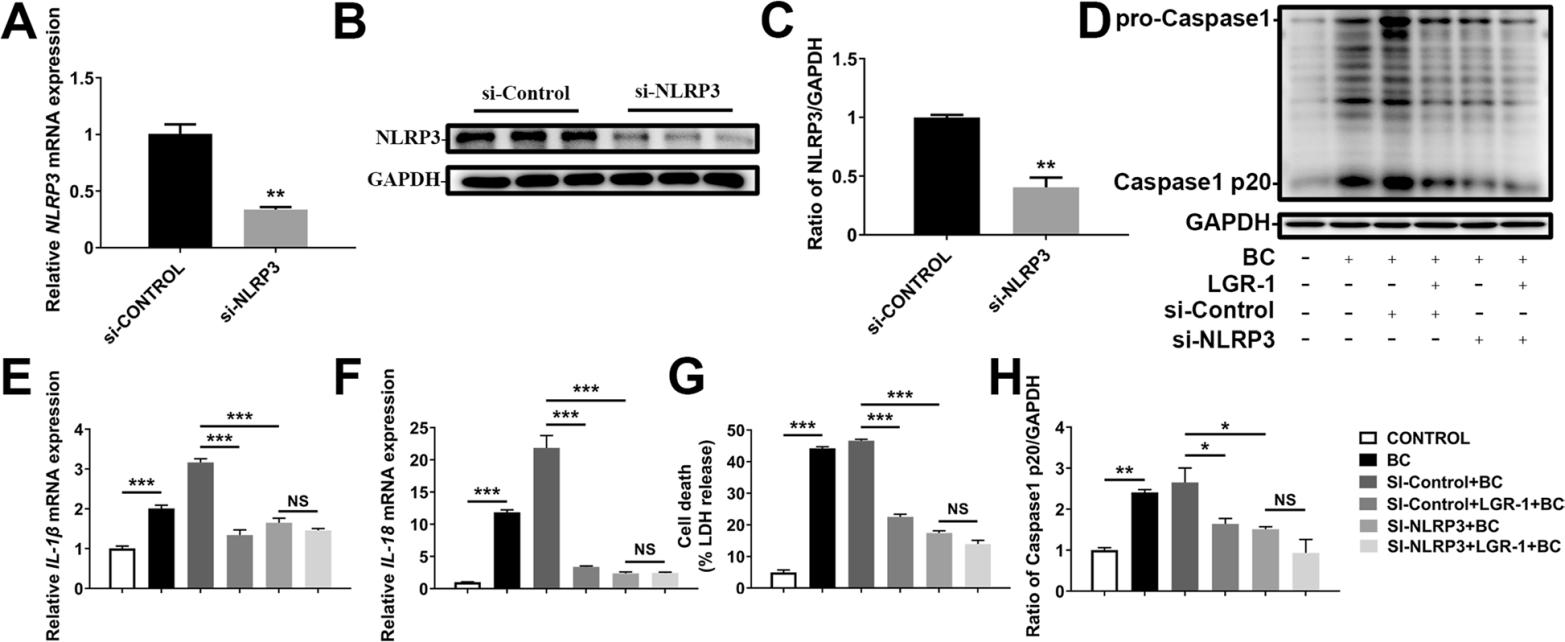
NLRP3 knockdown attenuated inflammatory responses. MAC-T cells were transfected with NLRP3 siRNA for 5 h and/or treated with BC (MOI = 5) and/or LGR-1 (MOI = 100) for another 3 h. (**A**) NLRP3 mRNA level. (**B**) Protein level of NLRP3. (**C**) Relative protein level of NLRP3. (**D**) Protein level of Caspase-1 p20. (**E**) *IL-1β* mRNA level. (**F**) *IL-18* mRNA level. (**G**) Cell death in MAC-T. (**H**) Relative protein level of Caspase1 p20. In **A** and **C**, data for the si-CONTROL group were used to normalize data of each treatment group. Comparisons among groups were calculated using the *t*-test. Data are means ± SEM of three independent experiments. In **E**–**H**, the data for the CONTROL group were used to normalize the data of each treatment group. Comparisons among groups were calculated using one-way ANOVA. Data are means ± SEM of three independent experiments. *P < 0.05, **P < 0.01, and ***P < 0.001

BC stimulation increased the protein expression of NLRP3, Caspase-1 p20, and GSDMD p30 (Fig. 6A and D–F, *P* < 0.05). Compared with the BC group, NLRP3 and Caspase-1 p20 decreased in the MCC950 (NLRP3 inhibitor) + BC group. Similarly, mRNA expression of *IL-1β* and *IL-18* increased in the BC group, but decreased in the MCC950 + BC group (Fig. 6B and C, *P*< 0.01). Compared with the si-CONTROL group, transfection with NLRP3 siRNA decreased the NLRP3 expression of mRNA and protein (Fig. 7A–C, *P* < 0.01). Compared with the CONTROL group, the protein expression of Caspase-1 p20 in the BC group was increased, while in the si-NLRP3 + BC group, it was significantly decreased (Fig. 7D and H, *P* < 0.01). Furthermore, compared with the CONTROL group, the mRNA abundances of *IL-1β* and *IL-18* in the BC group were significantly increased, whereas in the si-NLRP3 + BC group, they were significantly decreased (Fig. 7E and F, *P* < 0.001). Compared with the BC group, cell death in the si-NLRP3 + BC group was significantly decreased, which is consistent with the above changes in Caspase-1 and inflammatory cytokine abundance (Fig. 7G, *P*< 0.001).

## Discussion

*B. cereus* is often found in raw milk, and pasteurization cannot achieve complete sterilization. Therefore, the existence of *B. cereus* affects the shelf life of pasteurized milk [44]. As a food-borne pathogen, *B. cereus* can cause diarrheal and emetic syndromes through food entering the intestine, which adversely affects human health [9]. The main cause of disease is that *B. cereus* can produce a variety of virulence factors that play pathogenic roles. *B. cereus* was found to also produce pore-forming toxins, induce K^+^ efflux, and activate NLRP3 inflammasomes by forming pores, resulting in pyroptosis [14, 15]. When *B. cereus* infects the intestine, the intestinal microenvironment promotes the production of toxins, thereby damaging epithelial cells, lysing intestinal epithelial cells, and leading to diarrhea [45–47]. BC can secrete HBL, NHE, InhA, Hemolysin A, Hemolysin III, CytK, and EntFM, and has multi-drug resistance, rapid growth, and strong hemolysis properties. These properties impose great difficulties on the elimination and prevention of BC.

LGR-1 is currently the most studied female vaginal probiotic [27]. Previous studies have found that LGR-1 inhibits pathogen growth by adhering to mucosal surfaces [27, 48]. LGR-1 Lectin-like protein 1 binds to the glycosphingolipid GQ1 receptor on a wide variety of epithelial cells as well as important receptor bacterial lectins, suggesting its potential role in the adhesion capacity of LGR-1 [49]. LGR-1 can also inhibit pathogens by producing L-lactic acid, hydrogen peroxide, and bacteriocin-like compounds [50]. The particular lactic acid of this strain inhibits invasion and growth of bacteria and pathogens by reducing the pH level [51]. *Msp1* and *Msp2* genes were detected in LGR-1, which inhibit cell apoptosis induced by cytokines and protect intestinal epithelial barrier function [52]. The integrity of the epithelial lining is very important for the protection of human health. When it is disrupted, pathogens can enter tissues and the bloodstream, leading to inflammation and disease [28]. The tight junction is a key part of the epithelial barrier as it forms a permeability barrier and determines the selective permeability of epithelial cells [22]. Li et al. found that *Lactobacillus rhamnosus* SHA113 restored the damaged intestinal barrier as well as changes of the epithelial cell cytoskeleton caused by *E*. *coli* QBQ009, and also regulated the expression of tight junction proteins ZO-1 and occludin [53]. Similarly, after pre-treatment with *Lactobacillus plantarum*, the levels ZO-1 and occludin significantly increased around the tight junction structure, forming a paracellular seal between epithelial cells [29]. Further studies showed that *Lactobacillus plantarum* prevented *enteroinvasive E*. *coli*-induced protein expression and rearrangement of occludin and ZO-1 [30]. In this study, it was found that BC could destroy tight junctions and significantly down-regulate the expressions of ZO-1 and occludin, while pre-treatment with LGR-1 could offset this damage. This indicates that LGR-1 could effectively protect tight junctions and maintain the epithelial cell barrier.

NLRP3 is unique in the NLR receptor family because it is indirectly activated by numerous pathogenic and sterile inflammatory signals, including PAMPs of bacteria and viruses, pore-forming toxins, extracellular ATP, K^+^ efflux, and other signals [54]. Inflammasomes are important platforms for inflammatory signal transduction. When detecting body or tissue damage, inflammasomes release injury mediators to activate the inflammatory response [17]. NLRP3 inflammasome recruits contain caspase recruitment and activation domains ASC and Caspase-1, forming an inflammasome corpuscle complex [42]. Activation of NLRP3 leads to the cleavage of Caspase-1, the release of *IL-1β* and *IL-18*, as well as the cleavage of GSDMD and its transformation into an active form. Then, the active N-terminal fragment of GSDMD forms pores on the cell membrane, resulting in pyroptosis [13]. Cells infected by *B. cereus* show NLRP3 inflammasome activity [14, 15]. The expression levels of NLRP3 and ASC as well as the cleavages of Caspase-1 and GSDMD, *IL-1β*, and *IL-18* increased significantly after BC stimulation of MAC-T cells, indicating that BC activated NLRP3 inflammasome and caused pyroptosis. Similarly, Zhao et al. found that a virulent *B. cereus* strain from the deep-sea cold seep could lead to the activation of the NLRP3 inflammasome both in vivo and in vitro [55].

An abundance of evidence shows that LGR-1 can effectively inhibit NLRP3 inflammasome activity. Li et al. found that LGR-1 pretreatment eliminated activation of the NLRP3 inflammasome induced by *E*. *coli* [34]. Similarly, Wu et al*.* found that preincubation with LGR-1 effectively inhibited NLRP3 inflammasome and Caspase-1 activation caused by *E*. *coli* O111 infection in primary bovine mammary epithelial cells [31, 32]. In this study, it was found that pre-treatment with LGR-1 could effectively alleviate the increase of NLRP3, ASC, the cleavages of Caspase-1 and GSDMD, *IL-1β*, and *IL-18* caused by BC, while treatment with LGR-1 alone did not have any effect. LGR-1 could protect the structure of tight junctions and increase the expression of both ZO-1 and occludin, but it did not affect the activation of the NLRP3 inflammasome. This indicates that it reduced the stimulation of BC by protecting tight junctions, thereby weakening the activation of NLRP3 inflammasomes and reducing pyroptosis. Previous studies showed that LPS can destroy the integrity of the endothelial barrier, damage tight junctions, reduce the expressions of ZO-1 and occludin, and change the permeability of the cell barrier [56–58]. In this study, LPS + ATP was used to stimulate the NLRP3 inflammasome activity. Pretreatment with LGR-1 reduced the activation of NLRP3 inflammation and increased the expressions of ZO-1 and occludin, indicating that LGR-1 protects tight junctions and reduces the activation of the NLRP3 inflammasome.

Epithelial cells establish selectively permeable barriers to support cell nutrient absorption and waste secretion [59]. This selective permeable barrier is realized by the intercellular structure of tight junctions, which regulates the permeability of cells. When it is destroyed, permeation of toxins leads to inflammation [60]. Mathur et al. [14] and Fox et al. [15] found that the activation of NLRP3 inflammasome induced by virulence factors HBL and NHE of *B. cereus* required K^+^ efflux. It has also been shown that NHE induces pore formation, as it leads to the formation of conductive openings and penetrating cations [61, 62]. Furthermore, in this study, extracellular K^+^ was supplemented to inhibit the K^+^ efflux followed by BC stimulation. *IL-1β*, *IL-18*, and cell death decreased with increasing of K^+^ dose. Similarly, K^+^ also inhibited *IL-1β*, *IL-18*, and cell death after the stimulation of LPS + ATP. This suggests that LGR-1 prevented BC-induced K^+^ efflux by protecting tight junctions, which may also be one of the ways to prevent the activation of the NLRP3 inflammasome.

To prove that the NLRP3 inflammasome is activated after MAC-T cell infection by BC, pre-treatment with MCC950 (NLRP3 inhibitor) and knockdown of NLPR3 by si-NLRP3 were employed. NLRP3 expression was inhibited, thereby directly inhibiting the activation of the NLRP3 inflammasome. BC caused pyroptosis by activating the NLRP3 inflammasome, which is evidence for the decrease of Caspase1 p20 and GSDMD p30 after inhibition of NLRP3 by MCC950. Subsequently, si-NLRP3 was further used to silence NLRP3, showing that the expressions of inflammatory cytokines and cell death were significantly reduced after silencing. However, there was no significant difference in the expressions of inflammatory cytokines and cell death between the LGR-1 group and the BC group, indicating that LGR-1 did not affect MAC-T cells when NLRP3 had been silenced. Therefore, these results confirm that BC could activate the NLRP3 inflammasome to induce cell pyroptosis and LGR-1 could inhibit BC-induced activation of the NLRP3 inflammasome.

## Conclusion

**Fig. 8:**
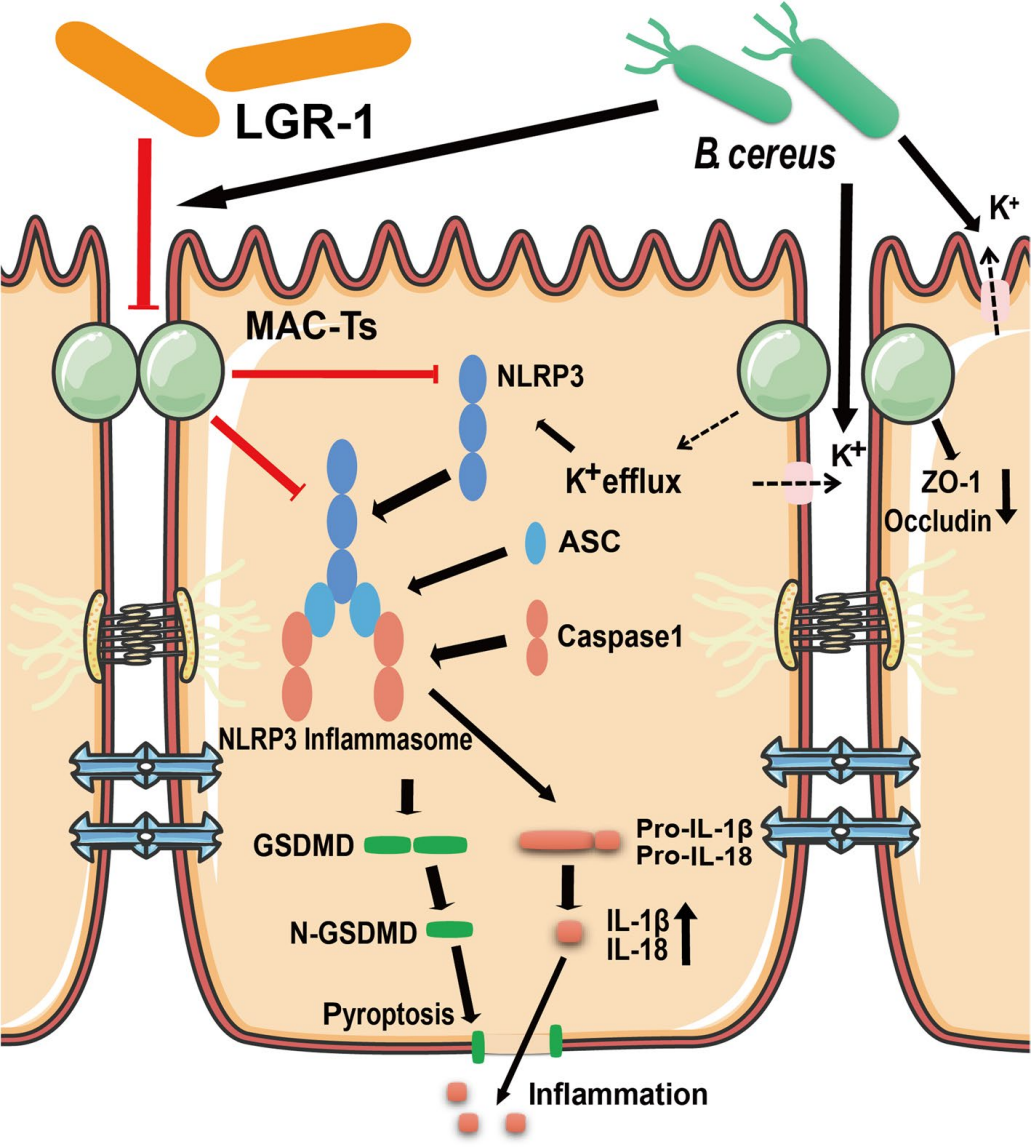
Schematic of the proposed model. LGR-1 can reduce BC-induced K^+^ efflux, inhibit the NLRP3 inflammasome activity, decrease the expressions of *IL-1β* and *IL-18*, reduce the production of active N-terminal fragment of GSDMD, and reduce both pyroptosis and the inflammatory response by protecting intercellular tight junctions. The solid black arrows indicate demonstrated effects, the dashed arrows indicate potential effects, red lines indicate negative or protective effects, and solid red arrows represent changes in expression

In summary, this experiment provides important evidence that LGR-1 protects intercellular tight junctions and reduces BC-induced activation of NLRP3 inflammasomes. Mechanisms of action include protecting intercellular tight junctions and inhibiting both the activation of NLRP3 inflammasome and pyroptosis (Fig. 8). The cytoprotective effect of LGR-1 at least partly depends on the protection of tight junctions. In this study, the probiotic LGR-1 prevented the further invasion of BC, which significantly harmed MAC-T cells isolated from dairy cow milk samples with mastitis. This may reduce mastitis caused by *B. cereus*, thereby reducing the potential harm of *B. cereus* to human transmission through milk.

## Supplementary information

**Additional file 1. Fig. S1. **LGR-1 protects BC-damaged intercellular tight junctions. MAC-T cells were treated with BC(MOI = 5) and/or LGR-1 (MOI = 100). (A) Expression of ZO-1 in cells measured by immunofluorescence; scale bar shown in the lower right corner. (B) *ZO-1* mRNA level. (C) Occludin mRNA level. The data of the CONTROL group were used to normalize the data of each treated group. Comparisons among groups were analyzed using one-way ANOVA. Data are means ± SEM of three independent experiments. *P < 0.05, **P < 0.01, and ***P < 0.001 

## Data Availability

All data generated or analyzed during this study are included in this published article.

## Abbreviations

*B*. *cereus*: *Bacillus cereus*
NLRP3: NOD-like receptor family member pyrin domain-containing protein
ASC: Apoptosis-associated speck-like protein, which contain caspase recruitment and activation domain
IL: Interleukin
GSDMD: Gasdermin D
LPS: Lipopolysaccharides
ATP: Adenosine 5'-triphosphate
ZO: Zonula occludens
LGR-1: *Lactobacillus **rhamnosus* GR-1
*E*. *coli*: *Escherichia coli*
MOI: Multiplicity of infection
MRS: De Man, Rogosa and Sharpe
MIC: Minimum inhibitory concentration
siRNA: Short interfering RNA
SEM: Scanning electron microscopy
TEM: Transmission electron microscopy
qPCR: Real-time quantitative PCR
