# Supplementary figures and images for: Lactobacillus rhamnosus GR-1 attenuates foodborne Bacillus cereus-induced NLRP3 inflammasome activity in bovine mammary epithelial cells by protecting intercellular tight junctions
Source: J Anim Sci Biotechnol. 2022 Sep 9;13:101. doi: 10.1186/s40104-022-00752-w (PMC9461272; doi:10.1186/s40104-022-00752-w)

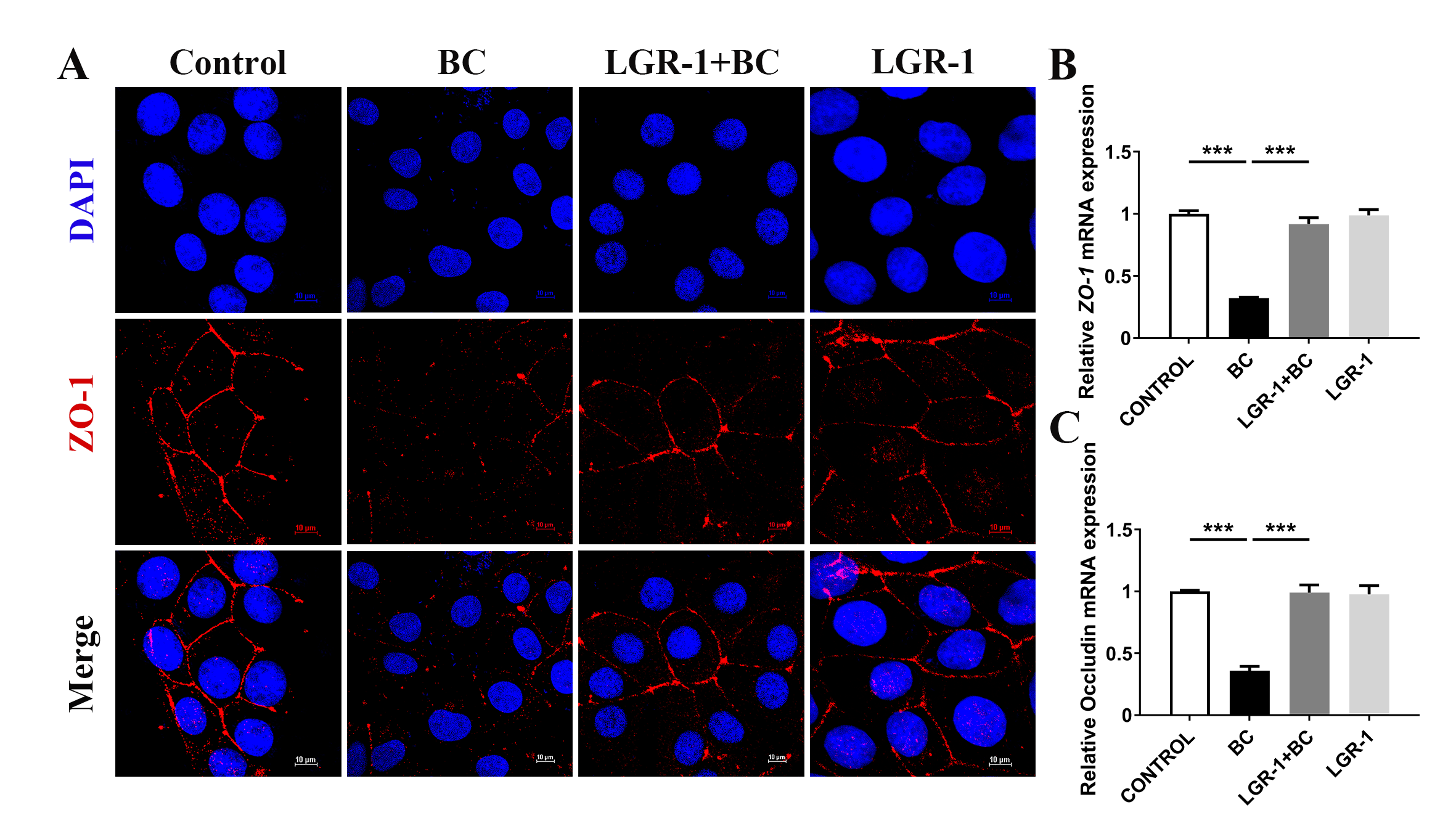

Supplement: Supplementary file 1 — Additional file 1. Fig. S1. LGR-1 protects BC-damaged intercellular tight junctions. MAC-T cells were treated with BC(MOI = 5) and/or LGR-1 (MOI = 100). (A) Expression of ZO-1 in cells measured by immunofluorescence; scale bar shown in the lower right corner. (B) ZO-1 mRNA level. (C) Occludin mRNA level. The data of the CONTROL group were used to normalize the data of each treated group. Comparisons among groups were analyzed using one-way ANOVA. Data are means ± SEM of three independent experiments. *P < 0.05, **P < 0.01, and ***P < 0.001 [file 40104_2022_752_MOESM1_ESM.tif]
